# Oxidation of Ceramic Materials Based on HfB_2_-SiC under the Influence of Supersonic CO_2_ Jets and Additional Laser Heating

**DOI:** 10.3390/ijms241713634

**Published:** 2023-09-04

**Authors:** Elizaveta P. Simonenko, Anatoly F. Kolesnikov, Aleksey V. Chaplygin, Mikhail A. Kotov, Mikhail Yu. Yakimov, Ilya V. Lukomskii, Semen S. Galkin, Andrey N. Shemyakin, Nikolay G. Solovyov, Anton S. Lysenkov, Ilya A. Nagornov, Artem S. Mokrushin, Nikolay P. Simonenko, Nikolay T. Kuznetsov

**Affiliations:** 1 Kurnakov Institute of General and Inorganic Chemistry of the Russian Academy of Sciences, Leninsky pr., 31, 119991 Moscow, Russia; il.nagornov.chem@gmail.com (I.A.N.); artyom.nano@gmail.com (A.S.M.); n_simonenko@mail.ru (N.P.S.); ntkuz@igic.ras.ru (N.T.K.); 2 Ishlinsky Institute for Problems in Mechanics of the Russian Academy of Sciences, 101-1 pr. Vernadskogo, 119526 Moscow, Russia; koles@ipmnet.ru (A.F.K.); chaplygin@ipmnet.ru (A.V.C.); kotoyarvi@list.ru (I.V.L.); galkin@ipmnet.ru (S.S.G.); solovyov@lantanlaser.ru (N.G.S.); 3 A. A. Baikov Institute of Metallurgy and Materials Science, Russian Academy of Sciences, Leninskii pr. 49, 119334 Moskow, Russia; toxa55@bk.ru

**Keywords:** UHTC, borides, SiC, supersonic carbon dioxide jet, laser heating, oxidation, induction HF-plasmatron

## Abstract

The features of oxidation of ultra-high-temperature ceramic material HfB_2_-30 vol.%SiC modified with 1 vol.% graphene as a result of supersonic flow of dissociated CO_2_ (generated with the use of high-frequency induction plasmatron), as well as under the influence of combined heating by high-speed CO_2_ jets and ytterbium laser radiation, were studied for the first time. It was found that the addition of laser radiation leads to local heating of the central region from ~1750 to ~2000–2200 °C; the observed temperature difference between the central region and the periphery of ~300–550 °C did not lead to cracking and destruction of the sample. Oxidized surfaces and cross sections of HfB_2_-SiC-C_G_ ceramics with and without laser heating were investigated using X-ray phase analysis, Raman spectroscopy and scanning electron microscopy with local elemental analysis. During oxidation by supersonic flow of dissociated CO_2_, a multilayer near-surface region similar to that formed under the influence of high-speed dissociated air flows was formed. An increase in surface temperature with the addition of laser heating from 1750–1790 to 2000–2200 °C (short term, within 2 min) led to a two to threefold increase in the thickness of the degraded near-surface area of ceramics from 165 to 380 microns. The experimental results indicate promising applications of ceramic materials based on HfB_2_-SiC as part of high-speed flying vehicles in planetary atmospheres predominantly composed of CO_2_ (e.g., Venus and Mars).

## 1. Introduction

Ultra-high-temperature ceramic materials (UHTC) based on ZrB_2_-SiC and HfB_2_-SiC are currently recognized as very promising for operation under extreme conditions, such as at temperatures above 2000 °C in an oxygen-containing atmosphere with simultaneous high ablative loading [1,2,3,4,5,6,7,8]. These materials are positioned as interesting from the practical point of view for manufacture of the most thermally loaded parts (e.g., sharp edges of wings and nose parts) of high-speed aircraft, which are subjected to aerodynamic heating up to temperatures of 2000–2500 °C; the smaller the radius of curvature, the greater the thermochemical effect on the material [9,10,11]. At the same time, ultra-high temperatures are concentrated in a very small area of the flying vehicle, and the temperature difference between the leading edge and the side surface can be up to 1000 degrees [2,12,13,14,15,16]. The high thermal conductivity of basic components such as zirconium and hafnium diborides (from 58 [17] to 95–145 W·m^−1^·K^−1^ [18,19,20] for ZrB_2_ and from 51 [17] to 103 [21] W·m^−1^·K^−1^ for HfB_2_) and the material as a whole (from 44 to 141 W·m^−1^·K^−1^ [1,19,22,23,24] depending on the composition of ZrB_2_(HfB_2_)-SiC) help to avoid material destruction. Relatively high oxidation resistance in an atmosphere containing oxygen for units of tens of minutes is one of the basic properties of ultra-high-temperature materials for aerospace applications. Ongoing studies have confirmed that the introduction of silicon carbide or refractory metal silicide into the composition of UHTCs based on ZrB_2_(HfB_2_) supports a significant increase in their resistance to oxidation at temperatures >1800–2000 °C, including under the influence of high-speed gas flows containing atomic oxygen (which is characteristic of aerodynamic heating by supersonic/hypersonic air flows) while maintaining increased thermal conductivity [16,25,26,27,28,29,30,31,32,33]. Modification of these ceramic materials with carbon materials (graphite, carbon fibers or carbon nanotubes), as shown in [34,35,36,37,38,39,40,41], makes it possible to slightly improve crack resistance and thermal shock resistance. In our previous studies, it was established [42,43,44] that due to the introduction of a small amount (1–2 vol.%) of graphene into the composition of HfB_2_-30 vol.%SiC material, there is an opportunity to reduce the degree of its degradation (thickness of the oxidized layer and rate of entrainment) by reducing the temperature that is reached at the surface under the same thermochemical effect of supersonic dissociated air flow, which is probably due to the increase in thermal conductivity.

Despite the obvious advantages of ultra-high temperature ceramic ZrB_2_(HfB_2_)-SiC, for some unclear reason, its application is considered almost exclusively under an air atmosphere, i.e., for high-speed (hypersonic) aircraft in the Earth’s atmosphere. At the same time, the use of waveriders, the geometry of requires a small edge radius, has long been proposed as a potential candidate for the exploration of Mars and Venus [45,46]. Studies of the behavior of ultra-high-temperature ceramics in non-air gas atmospheres are virtually non-existent in the literature. There are only single papers investigating the behavior of such materials in nitrogen [47,48,49,50] and argon–hydrogen plasma [51]. For ceramics based on the HfB_2_-SiC system, there are only two studies on the effects of high-speed flows of dissociated nitrogen [49,50]. At the same time, it will be necessary to provide even higher oxidation resistance for sharp-edged materials to work in the atmospheres of the mentioned planets, since it is known that the atmosphere of both Mars and Venus is dominated by CO_2_ (95–96% [52,53,54]), which should exert an even higher thermal load on parts due to the increased chemical component during high-speed motion [9]. During the dissociation of a gas stream consisting almost entirely of CO_2_, the amount of reactive atomic oxygen formed will be much higher than that formed during the dissociation of air, in which the oxygen content is 21%. A detailed analysis of the available literature has shown that there are no publications on the modeling and experimental study of the interaction of high-speed carbon dioxide gas flows with promising ZrB_2_(HfB_2_)-SiC ceramic materials simulating CO_2_-based atmospheric entry.

For the most accurate study of the material degradation/oxidation process as a result of aerodynamic heating, arc-jet and inductively coupled plasma facilities are used, which allow for convective heating of the sample surface. However, in some cases, it is important to add a highly radiant heat flux, which is very effectively provided by laser irradiation [55,56,57,58]. For example, in [55], a specimen made of the Buran orbital vehicle’s heat-shield tile material with a black low-catalytic coating was exposed to a subsonic pure nitrogen plasma jet and laser radiation.

The aim of the present work is to study the oxidation process of ultra-high-temperature HfB_2_-30 vol.%SiC ceramics modified with 1 vol.% graphene under the combined effect of supersonic CO_2_ plasma jet and radiation heating by ytterbium laser.

## 2. Results and Discussion

### 2.1. Thermochemical Effects of Supersonic Carbon Dioxide Flow and Combined Heating with Laser Irradiation on the Surface of HfB_2_-SiC-C_G_ Samples

Samples of ultra-high-temperature ceramics of material composition (HfB_2_-30%SiC)-1%C_G_) fixed in a vertical water-cooled model were immersed in a supersonic CO_2_ jet after its specified parameters (anode power, 60 kW; pressure in the test chamber, ~9.0–9.1–10^2^ Pa) had been established. Sample 1 was kept under these conditions for 14 min, while for sample 2 the central region was exposed to additional laser radiation for 2 min starting from the 11th min (the diameter of the laser-heated area was ~7 mm). After switching off the laser, CO_2_ plasma heating of sample 2 was continued for a further 2 min; the total exposure time was also 14 min.

**Figure 1:**
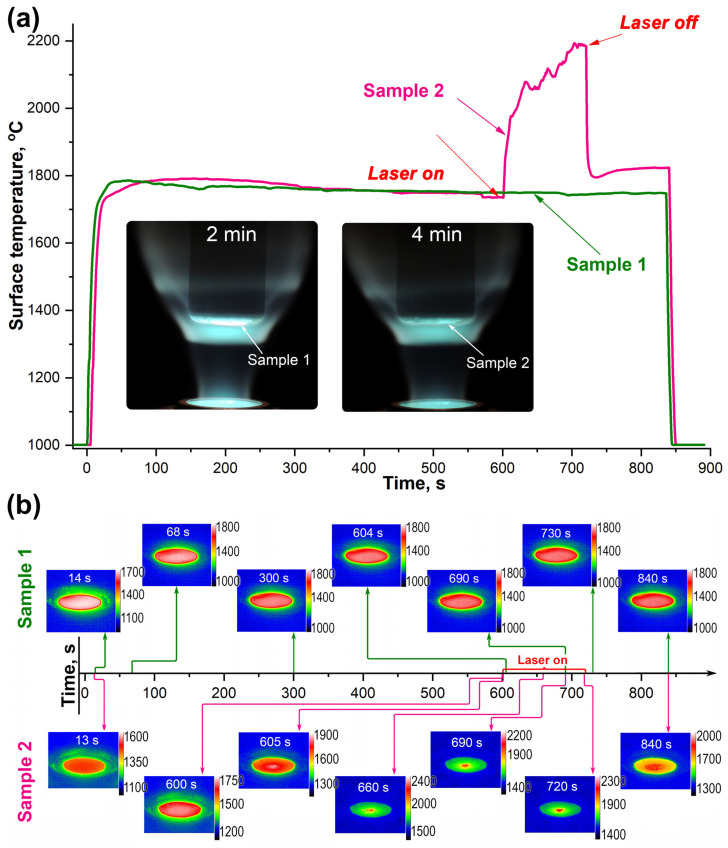
Variation of the average surface temperatures of samples 1 (green) and 2 (pink) from IR pyrometer data (**a**) (inset shows the appearance of the samples in a water-cooled model under CO_2_ plasma), as well as the temperature distribution (in °C) on the surface of these samples at specific moments of the test (**b**).

As can be seen in Figure 1a, until the beginning of the 11th minute, the average surface temperature of the samples was within the margin of error (since the exposure mode was identical), at 1750–1790 °C. After the addition of the laser heating component for sample 2, a sharp rise in the mean temperature to 2000–2200 °C was observed, with a tendency to increase with exposure time. When the laser was switched off, the surface temperature of sample 2 decreased but did not return to the initial value (that of sample 1 at the 13th minute of the test was 1745 °C) and was set at a higher level of ~1795–1810 °C. The surface temperature also exhibited a slight increase as the CO_2_ plasma exposure continued up to the value of 1820–1825 °C, which probably indicates the continuation of the process of formation of the barrier ceramic layer of low thermal conductive HfO_2_ and removal of silicate melt from the surface. Increasing the thickness of the hafnium oxide on the surface and in the near-surface region simultaneously with the removal of the SiO_2_-based melt leads to difficulties in dissipating the heat input into the sample volume (with a corresponding gradual heating of the surface). The complete evaporation of the silicate melt from the surface and the formation of a kind of “thermal barrier layer” of hafnium oxide, as shown in experiments on the influence of high-enthalpy air flows on ultra-high-temperature ceramic ZrB_2_(HfB_2_)-SiC, leads to a sharp increase in the surface temperature from ~1750–1850 °C (depending on the influence conditions) to 2300–2800 °C—the so-called “temperature jump” effect [25,28,43,59,60,61]. Presumably, this effect should also occur as a result of exposure to CO_2_ plasma at slightly different temperatures. In the present experiment, it was not observed, probably due to the relatively short exposure time (only 14 min) and lower heat fluxes.

Analysis of the temperature distribution over the frontal surface of the samples obtained from thermal imaging data (Figure 1b, top) allows us to state that sample 1, which was exposed exclusively to the high-speed flow of dissociated CO_2_ without laser heating, was maximally uniform over the whole period of the experiment. The temperature difference between the central region and the periphery did not change and was in the range of 70–110 °C. The maximum temperature (in the center of the sample) varied from 1765 to 1785 °C, while the minimum temperature (periphery) varied from 1665 to 1720 °C.

The temperature distribution on the surface of sample 2, which was subjected to complex heating, varied significantly depending on the exposure time (Figure 1b, bottom). During exposure to the supersonic CO_2_ flow at the beginning of the experiment, a uniform temperature distribution with a 50 °C difference from the center to the periphery was also observed, as for sample 1. However, within the first second of laser heating, a sharp difference between the temperatures of the central region, where the additional radiation heating was concentrated, with a temperature of ~2000 °C, and the periphery, with a temperature of ~1750–1800 °C, was observed. For the established mode of combined heating at 660–690 s of the experiment, the temperature difference was ~300–550 °C, with maximum temperatures in the center of up to ~2200–2300 °C and at the edge of the sample up to ~1750–1800 °C. At the same time, the visual and thermal imager showed a constant temperature change of local surface areas 0.2–0.3 mm in size, which is most likely related to the formation of bulge bubbles (results of intensive outgassing of SiC and HfB_2_ oxidation products, as well as evaporation of components of the protective borosilicate melt), which were further destroyed, while the walls of the burst bubbles were pressed by the flow and melted to the surface oxidized layer. It should be noted that the maximum temperatures ≥2200–2300 °C observed in some areas of the surface create the possibility of softening, melting, amorphization and submelting of refractory hafnium dioxide, especially in the presence of small admixtures of SiO_2_ [62].

After switching off the laser at 720 s of the test, the strong overheating of the central region of sample 2 disappeared, but the temperature difference in the time interval of 720–840 s was still significantly higher compared to sample 1: 150–200 °C compared to 75–110 °C, respectively. This is probably related to the increased roughness formed during overheating as a result of the formation of convexities due to intensive evaporation processes at sample surface temperatures of 2200–2300 °C.

After the heating was stopped, the surface temperature of both samples decreased to a value <1000 °C within 10–15 s (due to the atmospheric flow into the test chamber). The mass loss for the samples within the error limits is practically equal to 0.39% (sample 1) and 0.32% (sample 2).

### 2.2. Characteristics of Oxidation of HfB_2_-SiC-C_G_ Samples by Supersonic Flow of Carbon Dioxide and Additional Exposure to Laser Radiation

**Figure 2:**
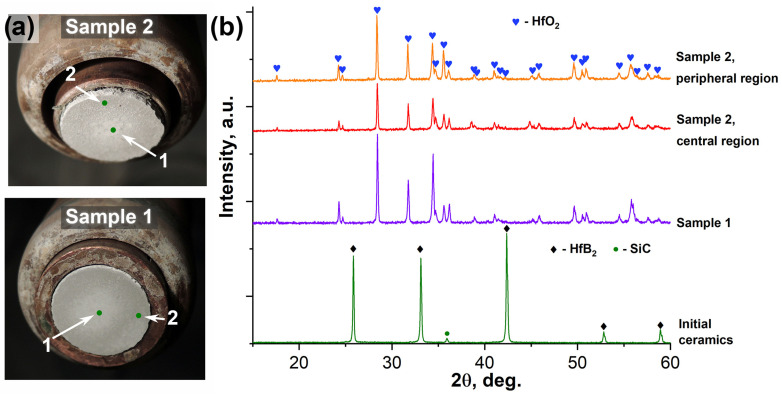
Appearance of samples in water-cooled copper model after cooling (**a**) and surface X-ray images before (green) and after thermochemical treatment (**b**): sample 1 (purple) and sample 2 in the central (red) and peripheral (orange) regions.

Figure 2a shows the appearance of samples 1 and 2 after completion of the thermochemical attack and cooling. Even at the macro level, the appearance of the oxidized surface of the samples differs significantly. For example, in sample 1, which was subjected to a more moderate load (the temperature did not exceed 1800 °C), the overall tone of the surface is grayer, which may indicate a shallower oxidation depth or a higher content of silicate glass in the oxidation products. In this case, the central area of sample 1 is whiter. At the same time, in sample 2, the central region appears lighter, also because it is smoother compared to the peripheral areas. Thus, the oxidized surface of sample 1 is rougher in the central region and a darker color compared to sample 2.

X-ray phase analysis (Figure 2b) shows that there are no reflections on the surface of the phases that make up the original ceramic samples (HfB_2_ [63] and SiC [64], as the graphene content is too low to show up in the X-ray diffraction patterns. The only crystalline oxidation product of both samples is monoclinic hafnium dioxide [65,66] in both the center and edge of the samples, irrespective of the steady-state temperatures at the surface during exposure. As the X-ray radiograms are displayed without intensity normalization, we can judge from their appearance that the central region of sample 2 is characterized by reduced intensities of all HfO_2_ reflections, i.e., we can note its lower crystallinity, probably due to the effect of high temperatures of up to 2200–2300 °C. For sample 1, the X-rays were recorded from the entire oxidized surface.

**Figure 3:**
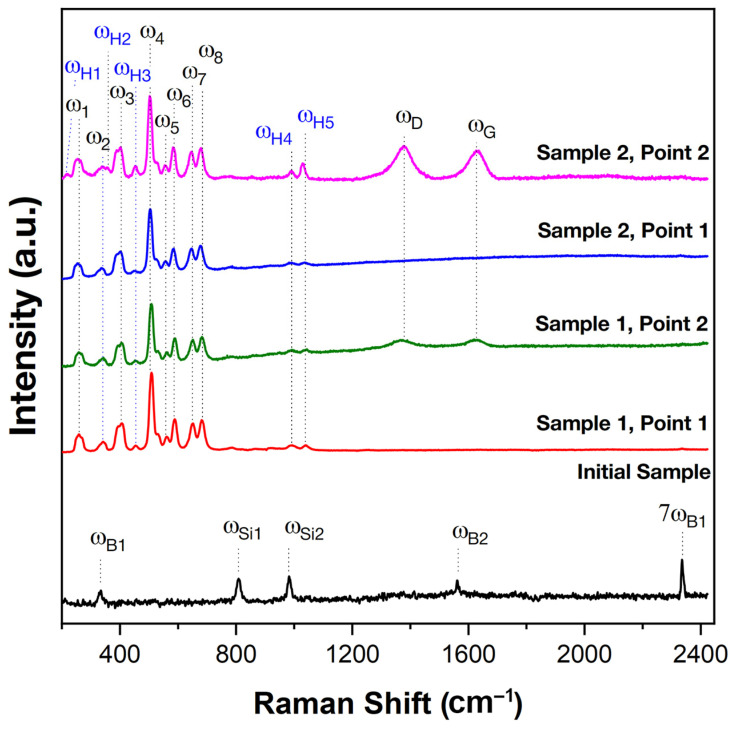
Raman spectra of the starting material, HfB_2_-SiC-C_G_ (black), and the oxidized surface of samples 1 and 2 in the central and peripheral regions (points 1 and 2, respectively, marked in Figure 2a).

Figure 3 shows the Raman spectra of the starting material, HfB_2_-SiC-C_G_, and the oxidized surface of the samples at points 1 and 2 (see Figure 2a). The starting material is characterized by five modes: ω_B1_, ω_Si1_, ω_Si2_, ω_B2_ and 7ω_B1_ at 334, 808, 981, 1561 and 2335 cm^−1^, respectively. The ω_B1_ and ω_B2_ bands correlate well with the characteristic modes of boron carbide [67,68], which could have formed as an impurity phase at the intergranular interface, while the 7ω_B1_ band is the seventh-order overtone of the ω_B1_ band. The ω_Si1_ and ω_Si2_ bands are characteristic modes of the silicon carbide phase (most likely polytype 3C) [69,70]. Raman spectra of HfB_2_-SiC-C_G_ samples show modes of silicon carbide and impurity B_4_C (the content of which is probably low, since its reflections are not found in X-ray diffraction patterns), whereas zirconium and hafnium diborides Raman modes are inactive [71,72].

After oxidation as a result of exposure to high-enthalpy CO_2_ jets, the surface spectra of the samples differ significantly from the initial spectra (Figure 3). The spectra obtained for all samples show a characteristic set of ω_1_–ω_8_ bands of the monoclinic phase of HfO_2_ at 256, 340, 401, 504, 557, 585, 645 and 681 cm^−1^, respectively, which correlates well with the A_g_ and B_g_ modes described in the literature [73,74,75], as well as weakly intense ω_H1_–ω_H5_ modes at 215, 357, 451, 990 and 1029 cm^−1^, respectively, belonging to the hafnon (HfSiO_4_) phase [76,77]. The spectra of the samples recorded at the periphery of the surface (at point 2) additionally show ω_D_ and ω_G_ at 1380 and 1635 cm^−1^, respectively, which are characteristic bands of different forms of carbon.

**Figure 4:**
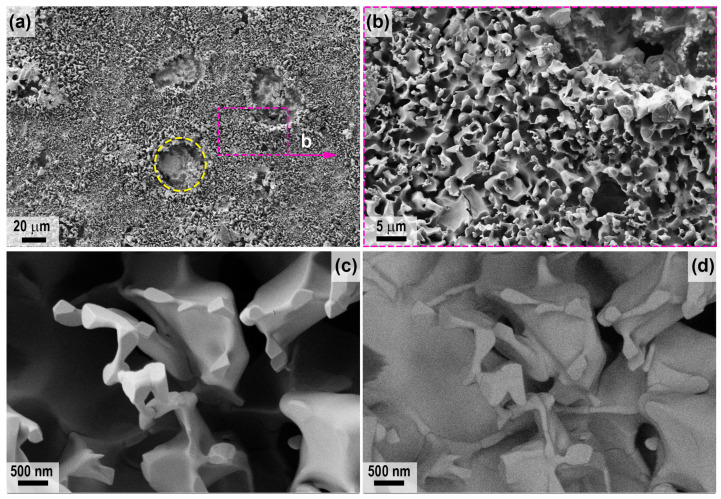
Microstructure of the oxidized surface of sample 1 in the central region according to SEM data: SE2 detector (**a**–**c**) in the medium atomic number contrast mode—ESB detector (**d**).

**Figure 5:**
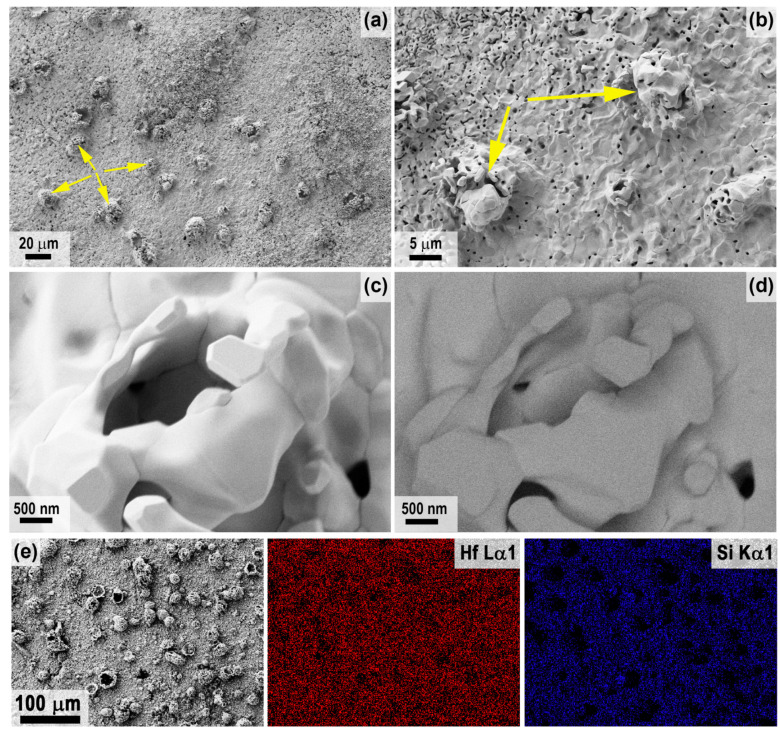
Microstructure of the oxidized surface at the edge of sample 1 from SEM data: SE2 detector (**a**–**c**) in mean atomic number contrast mode—ESB detector (**d**) and mapping of the distribution of Hf and Si atoms (**e**). Yellow arrows show examples of spherical bulges indicative of gassing processes.

A detailed study of the microstructure of the oxidized surface of sample 1 was carried out using SEM; the corresponding micrographs are shown in Figure 4 (center) and Figure 5 (periphery). It was found that the microstructure differed significantly in different areas. Thus, in the center (Figure 4), a very rough ceramic layer was formed on the basis of vertically oriented HfO_2_ particles of 2–3 μm in size, among which there are flat craters of 20–50 μm in diameter (example highlighted in the yellow oval in Figure 4a), probably traces of bubble fractures formed during the evaporation of SiO_2_ and B_2_O_3_. Figure 4d shows a micrograph of the surface area shown in Figure 4c taken with the ESB detector. There are significant foreign phase inclusions for vertically oriented HfO_2_ particles. X-ray microanalysis indicates the presence of only hafnium and oxygen and insignificant amounts of carbon in this area.

In the peripheral areas of sample 1, the oxidized ceramic layer is denser (Figure 5), comprising a cluster of flat particles 1–3 µm in size with pores of ~0.2–0.5 µm between them, among which there are spherical bulges up to 20 µm in diameter (examples are indicated by arrows in Figure 5a,b), representing traces of gas formation processes on the surface under the influence of the CO_2_ plasma. The denser structure of the oxidized layer can be explained by the fact that the predominant HfO_2_ particles are fused together by the residual silicate glass formed during the oxidation of the SiC, which did not have time to evaporate completely from the surface under the influence of temperatures of ~1700–1740 °C. This is confirmed by EDX data; in addition to Hf, O and C impurities, Si is also present in the peripheral areas, and as the mapping of Hf and Si distribution shows (Figure 5e), silicon is predominantly contained on the flat surface between the bubble protrusions. The atomic ratio of *n*(Hf):*n*(Si) at a distance of less than 1 mm from the edge of sample 1 is ~5.5:1.

**Figure 6:**
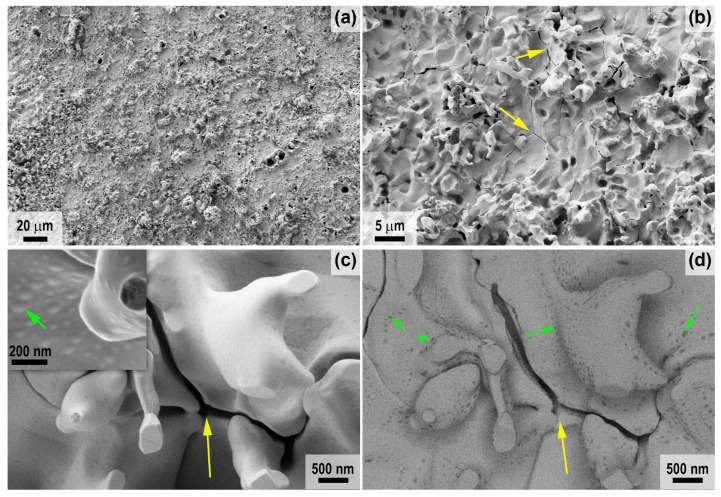
Microstructure of the oxidized surface of sample 2 in the central region according to SEM data: SE2 detector (**a**–**c**) in the medium atomic number contrast mode—ESB detector (**d**). Yellow arrows indicate cracks between particles, green arrows indicate bulges.

For sample 2, the difference in roughness of the oxidized surface in the central and peripheral regions is less pronounced, but it can be said that the surface layer is more friable compared to sample 1. The peculiarity of the microstructure of sample 2 (Figure 6, central region), in addition to the presence of significant porosity with a diameter of 1–10 μm (irregularly shaped pores up to 50–70 μm in size), is the presence of cracks between particles (yellow arrows in Figure 6), as well as the presence of small bulges (50–150 nm in diameter, green arrows in Figure 6c,d) on the surface of the HfO_2_ particles, which stand out when scanning in contrast mode by the average atomic number (Figure 6d). This is probably due to the presence of impurities of lighter atoms in these formations, such as silicon in the hafnon or silicate glass residues, as well as the non-stoichiometry in the composition of HfO_2_ or carbon impurity in its crystal lattice. However, elemental analysis by EDX does not allow the presence of silicon to be detected (probably less than 0.1%).

**Figure 7:**
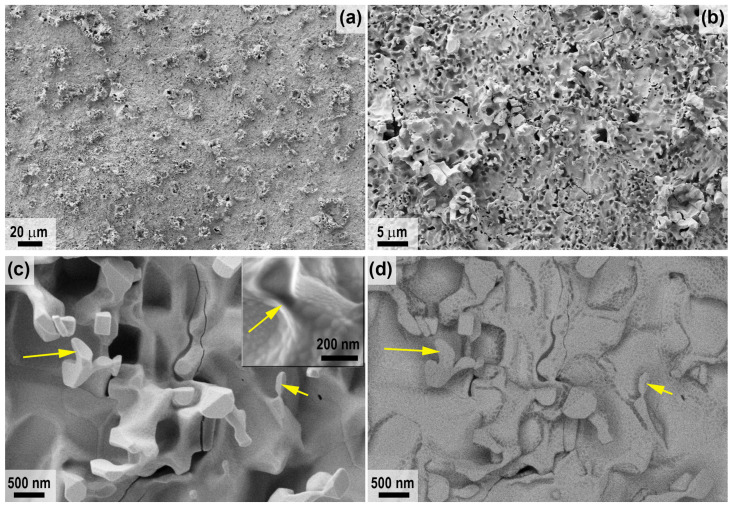
Microstructure of the oxidized surface at the edge of sample 2 from SEM data: SE2 detector (**a**–**c**) in average atomic number contrast mode with the ESB detector (**d**). Yellow arrows indicate vertically protruding HfO_2_ particles.

The peripheral regions of sample 2 have a very similar oxidized surface microstructure (Figure 7) but differ from the central region, with a less smoothed shape of vertically protruding HfO_2_ particles (marked with arrows in Figure 7c,d; Figure 7d represents the section of Figure 7c studied with the ESB detector). In contrast to the periphery of sample 1, elemental EDX analysis of sample 2 in these areas shows the presence of only hafnium and oxygen, with a small admixture of carbon.

It is very important to analyze the structure and elemental composition of the samples after exposure, which allows us to show the influence of the temperature set on the surface under the influence of a high-speed CO_2_ plasma flow or combined heating with the application of additional laser heating on the structure of the oxidized layer and the oxidation depth.

**Figure 8:**
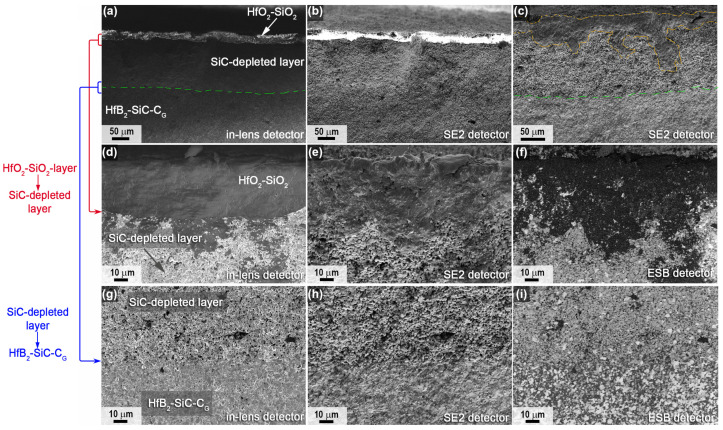
Cross-sectional microstructure of sample 1 (central region) according to SEM data: in-lens detector (**a**,**d**,**g**), SE2 detector (**b**,**c**,**e**,**h**), in contrast mode by average atomic number—ESB detector (**f**,**i**), acceleration voltage 1 kV (**a**,**c**–**i**), 20 kV (**b**).

For sample 1, which was subjected to milder exposure to a supersonic flow of dissociated CO_2_ only with a maximum surface temperature of 1785 °C, Figure 8 shows the microstructure of the spall in the central region. As can be seen, under the influence of the CO_2_ flow, a multilayer oxidized structure is formed, which is also typical for HfB_2_-SiC materials after exposure to a high-enthalpy dissociated air flow at a temperature >1750–1800 °C [25,60,78,79]. A relatively thin (~40 μm, Table 1) layer of hafnium oxide with a small admixture of SiO_2_, which remains after evaporation of the protective layer of silicate melt, is formed on the surface (Figure 8d,f). In addition, the thickness of the oxide layer increases to 105–110 μm in some areas.

Below this is a SiC-depleted region approximately 120 μm thick (Figure 8g–i, Table 1) formed by oxidation of silicon carbide at reduced oxygen levels by an active mechanism, which, in deeper layers, transitions rather abruptly to the unoxidized base material.

**Figure 9:**
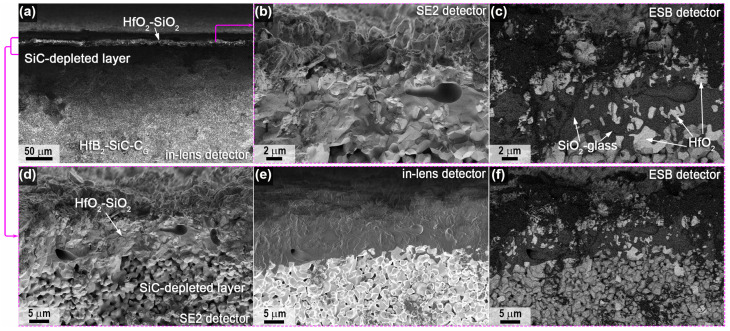
Cross-sectional microstructure of sample 1 (periphery) from SEM data: in-lens detector (**a**,**e**), SE2 detector (**b**,**d**), in contrast mode by mean atomic number—ESB detector (**c**,**f**), acceleration voltage 1 kV (**b**–**f**), 20 kV (**a**).

A similar structure is observed for the surface and near-surface regions at the periphery of sample 1 (Figure 9). A dense surface oxide layer transitions to a porous SiC-depleted layer. Among the differences, it is worth noting the reduced thickness of all layers and the fact that the transition from the SiC-depleted layer to the unoxidized HfB_2_-SiC-C_G_ material is diffuse, so its thickness presented in Table 1 is indicative.

**Figure 10:**
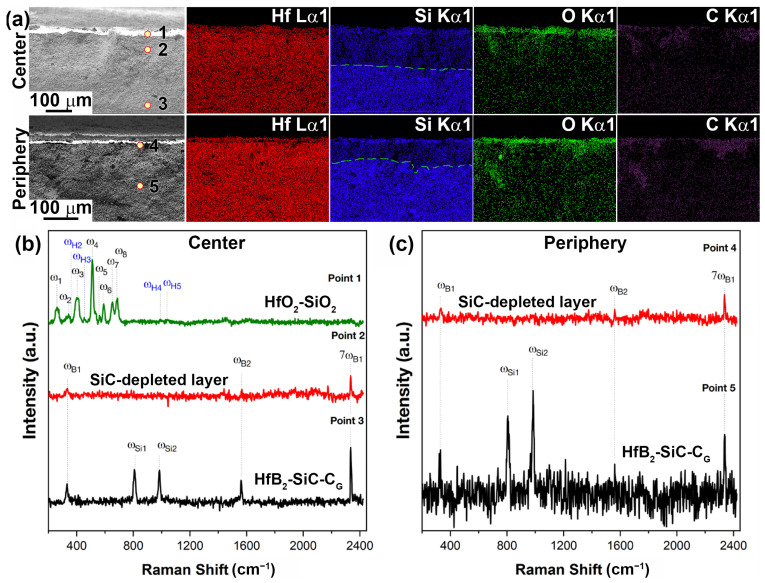
Mapping of Hf, Si, O and C distribution in the cross section of sample 1 in the central (top) and peripheral regions (bottom) (**a**), as well as Raman spectra in the indicated local regions (points 1–5) of chipped samples from the central (**b**) and peripheral parts of the sample (**c**).

The described distribution of oxide layers in the near-surface region of sample 1 is confirmed by the data mapping of the distribution of Hf, Si, O and C elements (the latter are provided as a reference due to the high error of their determination by the EDX method; Figure 10a). The figure for the peripheral region of sample 1 confirms a certain localization of silicon in the oxide region close to the surface, which is also found in the elemental analysis of the surface of this sample. In addition, despite the low reliability of the data on the distribution of carbon, we can speak with some approximation of its tendency to localize in the near-surface region. This is probably carbon sorbed from dissociated CO_2_ under the influence of CO radicals.

Figure 10b shows the Raman spectra of the spall from the central part of sample 1 at points 1–3 marked in Figure 10a. As can be seen, the region closest to the surface (point 1) is a set of bands corresponding to the surface. The spectra show characteristic Raman modes (ω_1_–ω_8_) of the monoclinic phase (HfO_2_) and even less intense modes (ω_H2–_ω_H5_) of the hafnon phase (HfSiO_4_). At point 2, corresponding to the SiC-depleted layer, the spectrum is significantly different; it lacks the modes of silicon carbide (confirming the EDX analysis data) and hafnium oxide, which could indicate that in this layer, the unoxidized HfB_2_ predominates, but only weakly intense bands (ω_B1,_ ω_B2_ and 7ω_B1_) are present, belonging to the impurity phase of boron carbide, probably on the surface of HfB_2_ grains. At point 3, in deeper layers (at 270–290 μm), a Raman spectrum is observed that completely repeats the original HfB_2_-SiC-C_G_ ceramic: five characteristic modes of the ω_B1_, ω_Si1_, ω_Si2,_ ω_B2_ and 7ω_B1_ phases of silicon carbide and boron carbide impurity are observed.

Raman spectra of the removed peripheral part of sample 1 subjected to thermochemical attack at temperature ≤1720 °C are shown in Figure 10c. In this zone, the oxide outer layer has a limited thickness (~12 µm, Table 1), which did not allow the Raman spectra to be correctly recorded on the instrument used. Therefore, the spectra only show points 4 and 5, corresponding to the SiC-depleted porous layer and deeper ceramic layers (≥135–140 μm). The data almost completely repeat the spectra obtained for the central part. In fact, only weak bands (ω_B1,_ ω_B2_ and 7ω_B1_) are present at point 4, which belong to the impurity phase of boron carbide. This means that a composition based mainly on Raman-inactive HfB_2_ can be assumed. The spectrum corresponding to point 5 shows characteristic modes of SiC and weak modes of the impurity, B_4_C.

**Figure 11:**
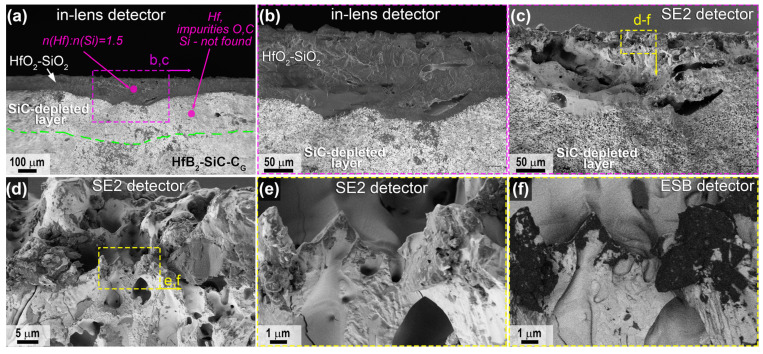
Cross-sectional microstructure of sample 2 in the central region according to SEM data: in-lens detector (**a**,**b**), SE2 detector (**c**–**e**), in medium atomic number contrast mode—ESB detector (**f**), acceleration voltage 1 kV (**b**–**f**), 20 kV (**a**).

**Figure 12:**
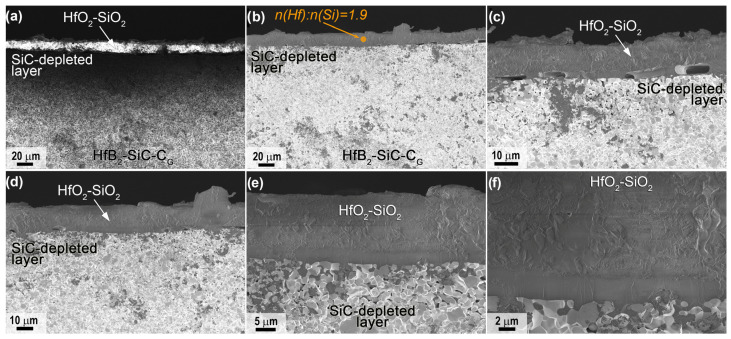
Cross-sectional microstructure of sample 2 (periphery) from SEM data (in-lens detector), with an acceleration voltage of 1 kV (**b**–**f**) and 20 kV (**a**).

**Figure 13:**
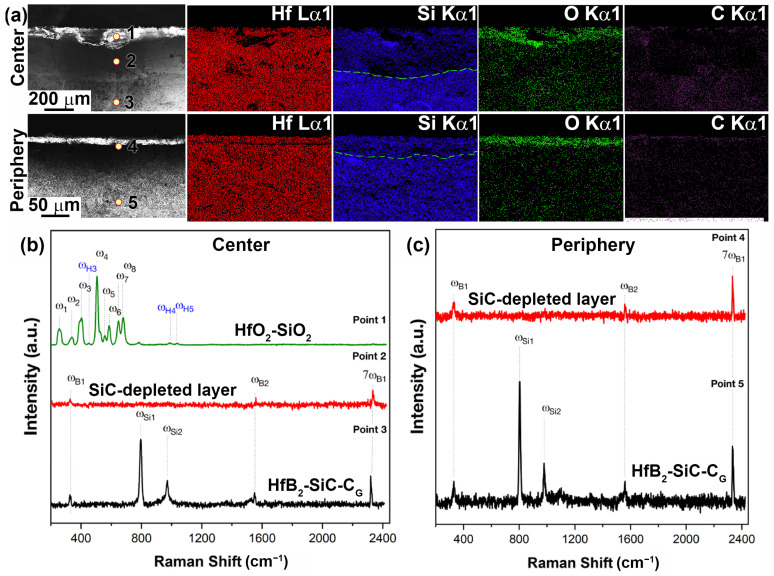
Mapping of Hf, Si, O and C distribution in the cross section of sample 2 in the central (top) and peripheral regions (bottom) (**a**), as well as Raman spectra in the indicated local regions (points 1–5) of the chipped samples from the central (**b**) and peripheral parts of the sample (**c**).

For sample 2, which was subjected to combined heating to much higher temperatures in the central region according to the SEM data, the distribution of the near-surface layers in the slip was similar (Figure 11, Figure 12 and Figure 13), but the thickness of both the HfO_2_-based oxide layer and the SiC-depleted layer was two to three times greater than for sample 1 (Table 1). For example, the thickness of the top oxide layer reached 140 μm (Figure 11b,c), and that of the SiC-depleted layer reached 230–250 μm. Note the much lower density of the oxide layer in the center of sample 2; in addition to rounded pores of 1–5 μm, there are bulk pores of up to 50–100 μm in the lower part oriented along the sample surface, indicating rapid and non-equilibrium gas release under thermochemical action. The ratio *n*(Hf):*n*(Si) in the volume of the oxide layer is 1.5, i.e., with the dominance of HfO_2_ as an oxidation product of HfB_2_, there is also a rather large amount of SiO_2_.

A very dense oxide layer with a thickness of ~20 μm was formed at the edge of sample 2 (Figure 12), in which large horizontally oriented pores were formed at the boundary with the porous SiC-depleted region, as also noted for the central region of the specimen but more evenly distributed across the surface of the specimen. The total thickness of the near-surface degradation region of the material was ~90 μm (Table 1), which is practically identical to that of sample 1.

Mapping of the distribution of the Hf, Si, O and C elements (Figure 13a) also confirms the formation of a multilayer near-surface region both in the center of sample 2, which was heated to ~2300 °C, and at the edge of the sample, whose temperature did not exceed 1800 °C. The Raman spectra (Figure 13b,c) for the labelled chipping points are also fully consistent with those for sample 1 (Figure 10b,c). Thus, the principal structure of the multilayer degraded near-surface region is essentially independent of the temperature formed at the surface as a result of the thermochemical effect of the supersonic flow of dissociated CO_2_ (including additional laser heating):

(1) External oxide layer based on HfO_2_ with admixture of HfSiO_4_ and, probably, silicate melt;
(2) SiC-depleted layer based on HfB_2_ with B_4_C admixture, which transitions to the unoxidized HfB_2_-SiC-C_G_ material.

However, the thickness of all components of the oxidized near-surface region and the porosity and defects of the outer oxide region are significantly dependent on the surface temperature.

## 3. Materials and Methods

### 3.1. Synthesis and Sample Preparation

Reagents: tetraethoxysilane (TEOS) Si(OC_2_H_5_)_4_ (>99.99%, EKOS-1 JSC, Moscow, Russia), LBS-1 Bakelite varnish (Karbolit OJSC, Moscow, Russia), formic acid CH_2_O_2_ (>99%, Spektr-Chem LLC, Moscow, Russia), hafnium diboride (>98%, particle size, 2–3 microns; aggregate size, ~20–60 microns; Tugoplavkie Materialy LLC, Taganrog, Russia) and graphene oxide powder (size of graphene flakes, ≤3 µm; thickness ≤ 2 graphene layers; “AkKo Lab” LLC, Moscow, Russia).

The preparation of the ceramic materials (HfB_2_-30%SiC)-1%C_G_) was carried out according to a previously described method [42,43,44] using the reactive hot pressing method, since reactive high-temperature consolidation allows us to significantly reduce the temperature required to obtain dense samples [80,81,82,83,84,85]. Briefly, using sol–gel technology, a composite powder, HfB_2_-SiO_2_-C, containing, in addition to carbon black, the required amount of graphene, was synthesized and used for hot pressing of ceramics in graphite molds using a Thermal Technology Inc. hot press (model HP20-3560-20) at a temperature of 1800 °C (heating rate, 10 °C/min; dwell time, 15 min) and a pressure of 30 MPa. As a result, cylindrical ceramic samples with a diameter of 15 mm, a thickness of ~3.9 mm and a relative density of 98% were produced. XRD data confirm the formation of cubic SiC, with the major phase being hexagonal HfB_2_. The particle size of the HfB_2_ is predominantly 2–5 µm, with inclusions of synthesized SiC no larger than 1–1.5 µm between them, as previously noted in [43,44].

### 3.2. Instrumentation

The oxidation resistance of the obtained material, (HfB_2_-30 vol.%SiC)-1 vol.%C_G_, under the influence of a supersonic flow of dissociated carbon dioxide was studied using a 100 kW high-frequency induction plasmatron VGU-4 [86,87] with a sonic nozzle with an outlet diameter of 30 mm. The distance from the nozzle to the sample was 30 mm, the CO_2_ flow rate was 2.4 g/s (controlled by a Bronkhorst MV-306 electronic flow meter, Bronkhorst High-Tech BV, Ruurlo, The Netherlands) and the chamber pressure was 9.1 ± 0.2 × 10^2^ Pa. The sample, in the form of a cylinder with a diameter of 15 mm and a thickness of ~3.9 mm, was immersed in the high-enthalpy jet at a plasmatron anode power (N) of 60 kW. Under these conditions, the sample was held until the end of the experiment; the total exposure time was 14 min (840 s).

Ceramic sample 2 was additionally exposed to laser radiation for 2 min starting from the 11th min of the experiment using an IPG Photonics YLPN-1-100-200-R pulsed fiber ytterbium laser (IRE-Polus, Fryazino, Moscow region, Russia) with a wavelength of 1.064 microns, with high directivity and stability of radiation, laser output power of 196 W, incident power of 168 W and a beam diameter of 7.1 mm. After switching off the laser, heating of the sample continued only due to the influence of the CO_2_ plasma jet of the plasmatron. The configuration of the test facility (HF plasmatron and ytterbium laser) is described in detail in [55]. The difference was that no lens was installed on the optical axis, which allowed us to achieve the maximum irradiation intensity.

The geometry of the water-cooled model in which the samples were mounted is described in detail in [49,88,89]. The samples were friction-mounted in the water-cooled calorimeter socket, with the gap filled with flexible SiC and carbon fiber insulation to minimize heat losses.

The measurement of the average temperature of the heated sample surface without laser radiation was carried out using a Mikron M770S infrared pyrometer in spectral ratio pyrometer mode (temperature range, 1000–3000 °C; diameter of the viewing area, ~5 mm in the central part of the sample, Mikron Infrared Inc., Oakland, CA, USA). The temperature distribution on the sample surface was studied using a Tandem VS-415U thermal imager (OOO «PK ELGORA», Korolev, Moscow region, Russia). Thermal images were recorded with the spectral emissivity vale (ε_λ_) set at a wavelength of 0.65 μm equal to 0.3, which was established as a result of preliminary experiments for fully oxidized HfB_2_-SiC samples in the CO_2_ flow. Furthermore, during the analysis of the thermal imaging data, the surface temperatures were corrected to the real ε_λ_ values if necessary. A correction factor of 0.93 for the transmittance of the quartz window of the test chamber was used to obtain the thermal images. Laser radiation at a wavelength of 1.064 μm affected the spectral ratio pyrometer data, so the only available tool to measure temperature during laser exposure was the thermal imager, with an operating wavelength of 0.65 μm. The temperature was determined from the thermal images as an average over an area equal to the pyrometer viewing area.

X-ray patterns of the obtained ceramic materials before and after exposure to supersonic carbon dioxide flow and after combined exposure to CO_2_ plasma and laser radiation, were recorded on a Bruker D8 Advance X-ray diffractometer (CuK_α_ radiation, and 0.02° resolution with signal accumulation in the point for 0.3 s, Bruker, Billerica, MA, USA). X-ray phase analysis was performed using MATCH!—Phase Identification from Powder Diffraction, Version 3.8.0.137 (Crystal Impact, Bonn, Germany), Crystallography Open Database (COD).

Raman spectra were recorded on a Confotec NR500 Raman spectrometer (20×/0.75 objective; 532 nm laser; grating: 600, SOL Instruments, Augsburg, Germany). The signal accumulation time was 60 s.

A study of the surface microstructure features of the obtained materials before and after exposure to a supersonic flow of CO_2_ dissociated was carried out by scanning electron microscopy (SEM) on a three-beam NVision 40 (Carl Zeiss, Oberkochen, Germany) work station with accelerating voltages of 1, 2 and 20 kV using secondary electron (SE2), energy-selective backscattered (ESB) and in-lens detectors, respectively. The elemental composition of the regions was determined using an Oxford Instruments energy-dispersive analysis device (EDX, Oxford, UK; accelerating voltage, 20 kV).

## 4. Conclusions

The features of oxidation of ultra-high-temperature ceramic material HfB_2_-30 vol.%SiC modified with a low (1 vol.%) amount of graphene under the influence of supersonic flow of dissociated CO_2_ and as a result of combined radiative and convective heating provided by additional application of ytterbium laser were studied. For sample 1, in which the influence of supersonic CO_2_ jets was investigated, with the chosen parameters of the experiment (CO_2_ flow rate, 2.4 g/s; chamber pressure, 9.1 ± 0.2 × 10^2^ Pa; plasmatron anode power, 60 kW; duration of influence, 14 min), a stabilization of the surface temperature at values of 1720–1785 °C was observed. There was no tendency of noticeable temperature increase, which could lead to a sharp temperature rise up to ~2200–2700 °C, known as temperature-jump phenomenon, as previously observed in similar samples under the influence of high-enthalpy air flows [12,25,28,43]. The total thickness of the near-surface degradation region (oxide layer plus SiC-depleted layer) varied from ~100 to ~165 μm for the peripheral and central regions, respectively.

It was found that the addition of laser irradiation leads to local heating of the central region of the sample from a temperature of ~1750 to ~2000–2200 °C (maximum temperature ~2300 °C). The resulting temperature difference from the central region to the edge, which is ~300–550 °C, did not lead to cracking or destruction of the sample. After switching off the laser, the surface temperature decreases to 1800–1825 °C, that is, there is no return to the temperature of the sample before the laser irradiation, probably due to changes in the microstructure, elemental composition and degree of crystallinity of the oxidized surface.

It was found that a change in the average surface temperature during thermochemical action from 1750–1790 to 2000–2200 °C (even briefly, within 2 min out of 14 min of total duration) leads to a sharp, two- to threefold increase in the thickness of the oxidized near-surface region. The formation of a multilayer degradation region typical of the oxidation of HfB_2_-SiC ceramics in air at temperatures >1750–1800 °C is observed. The upper layer of HfO_2_ with residual content of silicate melt passes into a porous SiC-depleted region (based on weakly oxidized HfB_2_), below which there is a zone of unoxidized material. 

In conclusion, the studies carried out in the present research demonstrate the promising application of HfB_2_-SiC-based ceramics in the composition of high-speed flight vehicles in an atmosphere composed mainly of CO_2_. The continuation and development of experiments to study the behavior of high-temperature ceramics based on ZrB_2_(HfB_2_)-SiC systems in complex gaseous environments based on CO_2_, N_2_ and their mixtures with air is an important and urgent task contributing to the creation of structural materials for space exploration.

## Figures and Tables

**Table 1:** Thickness of oxidized layers—top oxide (l_ox_), SiC-depleted layer (l_SiC-depl_) and total (l_Σ_)—in the central and peripheral regions of samples 1 and 2 as a function of maximum surface temperature during thermochemical treatment.

	Sample 1		Sample 2	
	Central Region	Peripheral Region	Central Region	Peripheral Region
t_max_, °C	1765–1785	1665–1720	2200–2300	1750–1800
l_ox_, µm	40 ± 20 *	12 ± 4	140 ± 25	20 ± 5
l_SiC-depl_, µm	120 ± 20	90 ± 10 **	230 ± 20	70 ± 10 **
l_Σ_, µm	165 ± 10	100 ± 10 **	380 ± 2	90 ± 10 **

* Oxide inclusions occur in cavities up to 105–110 µm deep. ** The transition from the SiC-depleted region to the unoxidized material is diffuse, so the presented data are indicative.

## Data Availability

Not applicable.

## References

[1] Nisar A., Hassan R., Agarwal A., Balani K. (2022). Ultra-High Temperature Ceramics: Aspiration to Overcome Challenges in Thermal Protection Systems. Ceram. Int..

[2] Savino R., Criscuolo L., Di Martino G.D., Mungiguerra S. (2018). Aero-Thermo-Chemical Characterization of Ultra-High-Temperature Ceramics for Aerospace Applications. J. Eur. Ceram. Soc..

[3] Simonenko E.P., Sevast’yanov D.V., Simonenko N.P., Sevast’yanov V.G., Kuznetsov N.T. (2013). Promising Ultra-High-Temperature Ceramic Materials for Aerospace Applications. Russ. J. Inorg. Chem..

[4] Aguirre T.G., Lamm B.W., Cramer C.L., Mitchell D.J. (2022). Zirconium-Diboride Silicon-Carbide Composites: A Review. Ceram. Int..

[5] Ni D., Cheng Y., Zhang J., Liu J.-X., Zou J., Chen B., Wu H., Li H., Dong S., Han J. (2022). Advances in Ultra-High Temperature Ceramics, Composites, and Coatings. J. Adv. Ceram..

[6] Liu G., Yan C., Jin H. (2022). Colloidal Processing of Complex-Shaped ZrB_2_-Based Ultra-High-Temperature Ceramics: Progress and Prospects. Materials.

[7] Sonber J.K., Murthy T.S.R.C., Majumdar S., Kain V. (2021). Processing of ZrB_2_- and HfB_2_-Based Ultra-High Temperature Ceramic Materials: A Review. Mater. Perform. Charact..

[8] Dan A., Basu B. (2020). Understanding Spectrally Selective Properties of Solar Absorbers. Energy Saving Coating Materials.

[9] Squire T.H., Marschall J. (2010). Material Property Requirements for Analysis and Design of UHTC Components in Hypersonic Applications. J. Eur. Ceram. Soc..

[10] Fahrenholtz W.G., Hilmas G.E. (2012). Oxidation of Ultra-High Temperature Transition Metal Diboride Ceramics. Int. Mater. Rev..

[11] He R.J., Zhang X.H., Hu P. (2012). Ablation Property of ZrB_2_-SiC Composite Sharp Leading Edges with Varying Radiuses of Curvature under Oxy-Acetylene Torch. Key Eng. Mater..

[12] Simonenko E.P., Simonenko N.P., Gordeev A.N., Papynov E.K., Shichalin O.O., Kolesnikov A.F., Avramenko V.A., Sevastyanov V.G., Kuznetsov N.T. (2018). Study of the Thermal Behavior of Wedge-Shaped Samples of HfB_2_–45 Vol % SiC Ultra-High-Temperature Composite in a High-Enthalpy Air Flow. Russ. J. Inorg. Chem..

[13] Monteverde F., Savino R. (2012). ZrB_2_-SiC Sharp Leading Edges in High Enthalpy Supersonic Flows. J. Am. Ceram. Soc..

[14] Jin X., He R., Zhang X., Hu P. (2013). Ablation Behavior of ZrB_2_–SiC Sharp Leading Edges. J. Alloys Compd..

[15] Sciti D., Savino R., Silvestroni L. (2012). Aerothermal Behaviour of a SiC Fibre-Reinforced ZrB_2_ Sharp Component in Supersonic Regime. J. Eur. Ceram. Soc..

[16] Bianco G., Nisar A., Zhang C., Boesl B., Agarwal A. (2023). Predicting Oxidation Damage of Ultra High-Temperature Carbide Ceramics in Extreme Environments Using Machine Learning. Ceram. Int..

[17] Samsonov G.V., Bolgar A.S., Guseva E.A., Klochkov L.A., Kovenskaya B.A., Serebryakova T.I., Timofeeva I.I., Turchanin A.G., Fesenko V.V. (1973). Thermophysical Properties of Transition Metal Carbides and Diborides. High Temp.-High Press..

[18] Kinoshita H., Otani S., Kamiyama S., Amano H., Akasaki I., Suda J., Matsunami H. (2001). Zirconium Diboride (0001) as an Electrically Conductive Lattice-Matched Substrate for Gallium Nitride. Jpn. J. Appl. Phys..

[19] Zhang L., Pejaković D.A., Marschall J., Gasch M. (2011). Thermal and Electrical Transport Properties of Spark Plasma-Sintered HfB_2_ and ZrB_2_ Ceramics. J. Am. Ceram. Soc..

[20] Guria J.F., Bansal A., Kumar V. (2021). Effect of Additives on the Thermal Conductivity of Zirconium Diboride Based Composites—A Review. J. Eur. Ceram. Soc..

[21] Opeka M.M., Talmy I.G., Wuchina E.J., Zaykoski J.A., Causey S.J. (1999). Mechanical, Thermal, and Oxidation Properties of Refractory Hafnium and Zirconium Compounds. J. Eur. Ceram. Soc..

[22] Weng L., Han W., Li X., Hong C. (2010). High Temperature Thermo-Physical Properties and Thermal Shock Behavior of Metal–Diborides-Based Composites. Int. J. Refract. Met. Hard Mater..

[23] Povolny S.J., Seidel G.D., Tallon C. (2022). Numerical Investigation of Thermomechanical Response of Multiscale Porous Ultra-High Temperature Ceramics. Ceram. Int..

[24] Potanin A.Y., Astapov A.N., Pogozhev Y.S., Rupasov S.I., Shvyndina N.V., Klechkovskaya V.V., Levashov E.A., Timofeev I.A., Timofeev A.N. (2021). Oxidation of HfB_2_–SiC Ceramics under Static and Dynamic Conditions. J. Eur. Ceram. Soc..

[25] Sevastyanov V.G., Simonenko E.P., Gordeev A.N., Simonenko N.P., Kolesnikov A.F., Papynov E.K., Shichalin O.O., Avramenko V.A., Kuznetsov N.T. (2015). Behavior of a Sample of the Ceramic Material HfB_2_-SiC (45 Vol %) in the Flow of Dissociated Air and the Analysis of the Emission Spectrum of the Boundary Layer above Its Surface. Russ. J. Inorg. Chem..

[26] Li N., Hu P., Zhang X., Liu Y., Han W. (2013). Effects of Oxygen Partial Pressure and Atomic Oxygen on the Microstructure of Oxide Scale of ZrB_2_–SiC Composites at 1500 °C. Corros. Sci..

[27] Scatteia L., Borrelli R., Cosentino G., Beche E., Sans J.-L., Balat-Pichelin M. (2006). Catalytic and Radiative Behaviors of ZrB_2_-SiC Ultrahigh Temperature Ceramic Composites. J. Spacecr. Rockets.

[28] Marschall J., Pejakovic D., Fahrenholtz W.G., Hilmas G.E., Panerai F., Chazot O. (2012). Temperature Jump Phenomenon during Plasmatron Testing of ZrB_2_-SiC Ultrahigh-Temperature Ceramics. J. Thermophys. Heat Transf..

[29] Nisar A., Zhang C., Boesl B., Agarwal A. (2020). A Perspective on Challenges and Opportunities in Developing High Entropy-Ultra High Temperature Ceramics. Ceram. Int..

[30] Bianco G., Nisar A., Zhang C., Boesl B., Agarwal A. (2022). A Critical Analysis of the Parameters Affecting the Oxidation Behavior of Ultra-high-temperature Diboride Ceramics. J. Am. Ceram. Soc..

[31] Zhao L., Hou C., Jin X., Li P., Wang Z., Fan X. (2023). Oxidation Behaviors of ZrB_2_–SiC Ceramics with Different Porosity. Adv. Eng. Mater..

[32] Sengupta P., Basu S., Manna I. (2023). Effect of TiC Reinforcement on Densification, Structural Evolution and High-Temperature Oxidation Behaviour of ZrB_2_-20 Vol Pct SiC Composite. Metall. Mater. Trans. A.

[33] Simonenko E.P., Simonenko N.P., Gordeev A.N., Kolesnikov A.F., Papynov E.K., Shichalin O.O., Tal’skikh K.Y., Gridasova E.A., Avramenko V.A., Sevastyanov V.G. (2018). Impact of a Supersonic Dissociated Air Flow on the Surface of HfB_2_–30 Vol % SiC UHTC Produced by the Sol–Gel Method. Russ. J. Inorg. Chem..

[34] Shahedi Asl M., Zamharir M.J., Ahmadi Z., Parvizi S. (2018). Effects of Nano-Graphite Content on the Characteristics of Spark Plasma Sintered ZrB_2_–SiC Composites. Mater. Sci. Eng. A.

[35] Shahedi Asl M., Ghassemi Kakroudi M. (2015). Characterization of Hot-Pressed Graphene Reinforced ZrB_2_–SiC Composite. Mater. Sci. Eng. A.

[36] An Y., Xu X., Gui K. (2016). Effect of SiC Whiskers and Graphene Nanosheets on the Mechanical Properties of ZrB_2_-SiCw-Graphene Ceramic Composites. Ceram. Int..

[37] Nisar A., Balani K. (2017). Phase and Microstructural Correlation of Spark Plasma Sintered HfB_2_-ZrB_2_ Based Ultra-High Temperature Ceramic Composites. Coatings.

[38] Gui K., Hu P., Hong W., Zhang X., Dong S. (2017). Microstructure, Mechanical Properties and Thermal Shock Resistance of ZrB_2_-SiC-C f Composite with Inhibited Degradation of Carbon Fibers. J. Alloys Compd..

[39] Arai Y., Inoue R., Goto K., Kogo Y. (2019). Carbon Fiber Reinforced Ultra-High Temperature Ceramic Matrix Composites: A Review. Ceram. Int..

[40] Chen Y. (2021). The Effects of Sintering Temperature on the Mechanical Properties and Toughening Mechanisms of Carbon Fibre-Reinforced HfB_2_-SiC Composites. Mater. Res. Express.

[41] Dubey S., Ariharan S., Nisar A., Saini S., Jana S.S., Wangaskar B., Das A., Khandekar S., Maiti T., Omar S. (2022). Domination of Phononic Scattering in Solid Solutioning and Interfaces of HfB_2_–ZrB_2_–SiC-Carbon Nanotube Based Ultra High Temperature Composites. Scr. Mater..

[42] Simonenko E.P., Simonenko N.P., Kolesnikov A.F., Chaplygin A.V., Sakharov V.I., Lysenkov A.S., Nagornov I.A., Kuznetsov N.T. (2022). Effect of 2 Vol % Graphene Additive on Heat Transfer of Ceramic Material in Underexpanded Jets of Dissociated Air. Russ. J. Inorg. Chem..

[43] Simonenko E.P., Simonenko N.P., Kolesnikov A.F., Chaplygin A.V., Lysenkov A.S., Nagornov I.A., Sevastyanov V.G., Kuznetsov N.T. (2021). Modification of HfB_2_–30% SiC UHTC with Graphene (1 Vol %) and Its Influence on the Behavior in a Supersonic Air Jet. Russ. J. Inorg. Chem..

[44] Simonenko E.P., Simonenko N.P., Kolesnikov A.F., Chaplygin A.V., Lysenkov A.S., Nagornov I.A., Simonenko T.L., Gubin S.P., Sevastyanov V.G., Kuznetsov N.T. (2022). Oxidation of Graphene-Modified HfB_2_-SiC Ceramics by Supersonic Dissociated Air Flow. J. Eur. Ceram. Soc..

[45] Anderson J.D., Lewis M.J., Kothari A.P., Corda S. (1991). Hypersonic Waveriders for Planetary Atmospheres. J. Spacecr. Rockets.

[46] Rodi P.E. (2021). Evaluation of the Capsule/Waverider Concept for Mars Entry, Descent, and Landing. Proceedings of the AIAA Aviation 2021 Forum.

[47] Monteverde F., Savino R. (2007). Stability of Ultra-High-Temperature ZrB_2_–SiC Ceramics under Simulated Atmospheric Re-Entry Conditions. J. Eur. Ceram. Soc..

[48] Savino R., De Stefano Fumo M., Silvestroni L., Sciti D. (2008). Arc-Jet Testing on HfB_2_ and HfC-Based Ultra-High Temperature Ceramic Materials. J. Eur. Ceram. Soc..

[49] Kolesnikov A.F., Kuznetsov N.T., Murav’eva T.I., Nagornov I.A., Sakharov V.I., Sevastyanov V.G., Simonenko E.P., Simonenko N.P., Chaplygin A.V., Shcherbakova O.O. (2022). Investigation of Heat Transfer to HfB_2_-SiC-Based Ceramics in Underexpanded Dissociated-Nitrogen Jets and Analysis of the Surface. Fluid Dyn..

[50] Simonenko E.P., Simonenko N.P., Kolesnikov A.F., Chaplygin A.V., Lysenkov A.S., Nagornov I.A., Mokrushin A.S., Kuznetsov N.T. (2022). Investigation of the Effect of Supersonic Flow of Dissociated Nitrogen on ZrB_2_–HfB_2_–SiC Ceramics Doped with 10 Vol.% Carbon Nanotubes. Materials.

[51] Alosime E.M., Alsuhybani M.S., Almeataq M.S. (2021). The Oxidation Behavior of ZrB_2_-SiC Ceramic Composites Fabricated by Plasma Spray Process. Materials.

[52] Moroz V.I. (1998). Chemical Composition of the Atmosphere of Mars. Adv. Sp. Res..

[53] Oyama V.I., Carle G.C., Woeller F., Pollack J.B., Reynolds R.T., Craig R.A. (1980). Pioneer Venus Gas Chromatography of the Lower Atmosphere of Venus. J. Geophys. Res..

[54] Marcq E., Mills F.P., Parkinson C.D., Vandaele A.C. (2018). Composition and Chemistry of the Neutral Atmosphere of Venus. Space Sci. Rev..

[55] Chaplygin A., Kotov M., Yakimov M., Lukomskii I., Galkin S., Kolesnikov A., Shemyakin A., Solovyov N. (2022). Combined Surface Heating by Laser Beam and Subsonic Nitrogen Plasma Jet. Fluids.

[56] Cushman G., Alunni A., Balboni J., Zell P., Hartman J., Empey D. (2018). The Laser Enhanced Arc-Jet Facility (LEAF-Lite): Simulating Convective and Radiative Heating with Arc-Jets and Multiple 50-KW CW Lasers. Proceedings of the 2018 Joint Thermophysics and Heat Transfer Conference.

[57] Gokcen T., Alunni A. (2019). CFD Simulations of the IHF Arc-Jet Flow: 9-Inch Nozzle, Flow Surveys, LEAF Wedge Calibration Data. Proceedings of the AIAA Aviation 2019 Forum.

[58] Momozawa A., Yokote N., Terutsuki D., Komurasaki K. (2021). Dynamic Oxidation of SiC with Arc-Heated Plasma Wind Tunnel and Laser Heating. Vacuum.

[59] Mungiguerra S., Cecere A., Savino R., Saraga F., Monteverde F., Sciti D. (2021). Improved Aero-Thermal Resistance Capabilities of ZrB_2_-Based Ceramics in Hypersonic Environment for Increasing SiC Content. Corros. Sci..

[60] Parthasarathy T.A., Rapp R.A., Opeka M., Cinibulk M.K. (2012). Modeling Oxidation Kinetics of SiC-Containing Refractory Diborides. J. Am. Ceram. Soc..

[61] Simonenko E.P., Gordeev A.N., Simonenko N.P., Vasilevskii S.A., Kolesnikov A.F., Papynov E.K., Shichalin O.O., Avramenko V.A., Sevastyanov V.G., Kuznetsov N.T. (2016). Behavior of HfB_2_-SiC (10, 15, and 20 Vol %) Ceramic Materials in High-Enthalpy Air Flows. Russ. J. Inorg. Chem..

[62] Shin D., Arróyave R., Liu Z.-K. (2006). Thermodynamic Modeling of the Hf–Si–O System. Calphad.

[63] Holleck H. (1967). Legierungsverhalten von HfB_2_ Mit Uran- Und Übergangsmetalldiboriden. J. Nucl. Mater..

[64] Wyckoff R.W.G. (1963). Second Edition. Interscience Publishers, New York, New Yorkrocksalt Structure. Cryst. Struct..

[65] Whittle K.R., Lumpkin G.R., Ashbrook S.E. (2006). Neutron Diffraction and MAS NMR of Cesium Tungstate Defect Pyrochlores. J. Solid State Chem..

[66] Ruh R., Corfield P.W.R. (1970). Crystal Structure of Monoclinic Hafnia and Comparison with Monoclinic Zirconia. J. Am. Ceram. Soc..

[67] Werheit H., Leithe-Jasper A., Tanaka T., Rotter H.W., Schwetz K.A. (2004). Some Properties of Single-Crystal Boron Carbide. J. Solid State Chem..

[68] Guo J., Zhang L., Fujita T., Goto T., Chen M. (2010). Pressure-Induced Depolarization and Resonance in Raman Scattering of Single-Crystalline Boron Carbide. Phys. Rev. B.

[69] Nakashima S., Harima H. (1997). Raman Investigation of SiC Polytypes. Phys. Status Solidi.

[70] Ghosh D., Subhash G., Orlovskaya N. (2008). Measurement of Scratch-Induced Residual Stress within SiC Grains in ZrB_2_–SiC Composite Using Micro-Raman Spectroscopy. Acta Mater..

[71] Shafiq M., Subhash G. (2014). A Novel Technique for the Determination of Surface Biaxial Stress under External Confinement Using Raman Spectroscopy. Exp. Mech..

[72] Stadelmann R., Hughes B., Orlovskaya N., Grasso S., Reece M.J. (2015). 2D Raman Mapping and Thermal Residual Stresses in SiC Grains of ZrB_2_–SiC Ceramic Composites. Ceram. Int..

[73] Tkachev S.N., Manghnani M.H., Niilisk A., Aarik J., Mändar H. (2005). Raman and Brillouin Scattering Spectroscopy Studies of Atomic Layer-Deposited ZrO_2_ and HfO_2_ Thin Films. Spectrochim. Acta Part A Mol. Biomol. Spectrosc..

[74] Wu R., Zhou B., Li Q., Jiang Z., Wang W., Ma W., Zhang X. (2012). Elastic and Vibrational Properties of Monoclinic HfO_2_ from First-Principles Study. J. Phys. D Appl. Phys..

[75] Zhou B., Shi H., Zhang X.D., Su Q., Jiang Z.Y. (2014). The Simulated Vibrational Spectra of HfO_2_ Polymorphs. J. Phys. D. Appl. Phys..

[76] Manoun B., Downs R.T., Saxena S.K. (2006). A High-Pressure Raman Spectroscopic Study of Hafnon, HfSiO_4_. Am. Mineral..

[77] Grüneberger A.M., Schmidt C., Jahn S., Rhede D., Loges A., Wilke M. (2016). Interpretation of Raman Spectra of the Zircon–Hafnon Solid Solution. Eur. J. Mineral..

[78] Li J., Lenosky T.J., Först C.J., Yip S. (2008). Thermochemical and Mechanical Stabilities of the Oxide Scale of ZrB_2_+SiC and Oxygen Transport Mechanisms. J. Am. Ceram. Soc..

[79] Han J., Hu P., Zhang X., Meng S., Han W. (2008). Oxidation-Resistant ZrB_2_–SiC Composites at 2200 °C. Compos. Sci. Technol..

[80] Shapkin N.P., Papynov E.K., Shichalin O.O., Buravlev I.Y., Simonenko E.P., Simonenko N.P., Zavjalov A.P., Belov A.A., Portnyagin A.S., Gerasimenko A.V. (2021). Spark Plasma Sintering-Reactive Synthesis of SiC and SiC–HfB_2_ Ceramics Based on Natural Renewable Raw Materials. Russ. J. Inorg. Chem..

[81] Simonenko E.P., Simonenko N.P., Papynov E.K., Shichalin O.O., Belov A.A., Nagornov I.A., Gorobtsov P.Y., Kuznetsov N.T. (2022). Effect of Nanocrystalline SiC Addition on Reactive SPS and Oxidation Resistance of Ta_4_HfC_5_ Ceramics. Ceram. Int..

[82] Simonenko E.P., Simonenko N.P., Papynov E.K., Gridasova E.A., Sevastyanov V.G., Kuznetsov N.T. (2018). Production of HfB_2_–SiC (10–65 Vol % SiC) Ultra-High-Temperature Ceramics by Hot Pressing of HfB_2_–(SiO_2_–C) Composite Powder Synthesized by the Sol–Gel Method. Russ. J. Inorg. Chem..

[83] Simonenko E.P., Simonenko N.P., Gordeev A.N., Kolesnikov A.F., Sevastyanov V.G., Kuznetsov N.T. (2019). Behavior of HfB_2_–30 Vol% SiC UHTC Obtained by Sol–Gel Approach in the Supersonic Airflow. J. Sol-Gel Sci. Technol..

[84] Simonenko E.P., Simonenko N.P., Gordeev A.N., Kolesnikov A.F., Chaplygin A.V., Lysenkov A.S., Nagornov I.A., Sevastyanov V.G., Kuznetsov N.T. (2021). Oxidation of HfB_2_-SiC-Ta_4_HfC_5_ Ceramic Material by a Supersonic Flow of Dissociated Air. J. Eur. Ceram. Soc..

[85] Papynov E.K., Portnyagin A.S., Modin E.B., Mayorov V.Y., Shichalin O.O., Golikov A.P., Pechnikov V.S., Gridasova E.A., Tananaev I.G., Avramenko V.A. (2018). A Complex Approach to Assessing Porous Structure of Structured Ceramics Obtained by SPS Technique. Mater. Charact..

[86] Gordeev A. Overview of Characteristics and Experiments in IPM Plasmatrons. VKI, RTO AVT/VKI Special Course on Measurement Techniques for High Enthalpy Plasma Flows. https://apps.dtic.mil/sti/citations/ADP010736.

[87] Chaplygin A.V., Vasil’evskii S.A., Galkin S.S., Kolesnikov A.F. (2022). Thermal State of Uncooled Quartz Discharge Channel of Powerful High-Frequency Induction Plasmatron. Phys. Kinet. Gas Dyn..

[88] Kolesnikov A.F., Lukomskii I.V., Sakharov V.I., Chaplygin A.V. (2021). Experimental and Numerical Modeling of Heat Transfer to Graphite Surface in Underexpanded Dissociated-Nitrogen Jets. Fluid Dyn..

[89] Lukomskii I.V., Chaplygin A.V., Kolesnikov A.F. (2021). A Device for Measuring the Heat Flux to the Surface of a Material Heated in a Jet of High-Enthalpy Gas to a High Temperature. Patent RU.

